# The Structure of a Rigorously Conserved RNA Element within the SARS Virus Genome

**DOI:** 10.1371/journal.pbio.0030005

**Published:** 2004-12-28

**Authors:** Michael P Robertson, Haller Igel, Robert Baertsch, David Haussler, Manuel Ares, William G Scott

**Affiliations:** **1**The Center for the Molecular Biology of RNA, University of CaliforniaSanta Cruz, CaliforniaUnited States of America; **2**Department of Chemistry and Biochemistry, University of CaliforniaSanta Cruz, CaliforniaUnited States of America; **3**Department of Molecular, Celland Developmental Biology, University of California, Santa Cruz, CaliforniaUnited States of America; **4**Howard Hughes Medical Institute and Department of Biomolecular Engineering, University of CaliforniaSanta Cruz, CaliforniaUnited States of America; University of WisconsinUnited States of America

## Abstract

We have solved the three-dimensional crystal structure of the stem-loop II motif (s2m) RNA element of the SARS virus genome to 2.7-Å resolution. SARS and related coronaviruses and astroviruses all possess a motif at the 3′ end of their RNA genomes, called the s2m, whose pathogenic importance is inferred from its rigorous sequence conservation in an otherwise rapidly mutable RNA genome. We find that this extreme conservation is clearly explained by the requirement to form a highly structured RNA whose unique tertiary structure includes a sharp 90° kink of the helix axis and several novel longer-range tertiary interactions. The tertiary base interactions create a tunnel that runs perpendicular to the main helical axis whose interior is negatively charged and binds two magnesium ions. These unusual features likely form interaction surfaces with conserved host cell components or other reactive sites required for virus function. Based on its conservation in viral pathogen genomes and its absence in the human genome, we suggest that these unusual structural features in the s2m RNA element are attractive targets for the design of anti-viral therapeutic agents. Structural genomics has sought to deduce protein function based on three-dimensional homology. Here we have extended this approach to RNA by proposing potential functions for a rigorously conserved set of RNA tertiary structural interactions that occur within the SARS RNA genome itself. Based on tertiary structural comparisons, we propose the s2m RNA binds one or more proteins possessing an oligomer-binding-like fold, and we suggest a possible mechanism for SARS viral RNA hijacking of host protein synthesis, both based upon observed s2m RNA macromolecular mimicry of a relevant ribosomal RNA fold.

## Abstract 2

The SARS RNA genome contains a unique structure that resembles a portion of ribosomal RNA; this may allow the virus to hijack its hosts protein synthesis machinery

## Introduction

**Figure 1:**
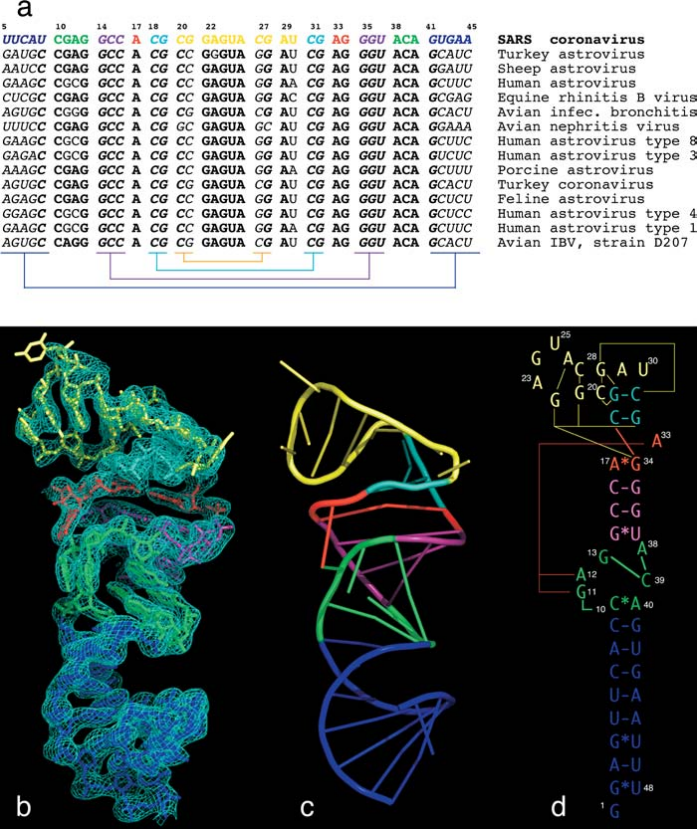
The Primary, Secondary, and Tertiary Structures of the SARS s2m RNA (A) Phylogenetic comparisons of s2m RNA sequences from various coronavirus and astrovirus species. The SARS RNA sequence is color-coded to match the color scheme used throughout. Conserved sequences are highlighted as bold letters, and co-varying sequences involved in conventional RNA helical base-pairing are indicated in italics. Sequence complements are indicated using color-coded brackets. (B) The 2.7-Å experimental SIRAS platinum-phased and solvent-flattened electron density map contoured at 1.25 root mean square deviation. The map allowed unambiguous tracing of the RNA molecule because the density was unambiguous for all backbone atoms and all nucleotide bases except U(25), U(30), and U(48). (C) A corresponding ribbon diagram highlighting the unusual fold. (D) Schematic representation of the s2m RNA secondary structure, with tertiary structural interactions indicated as long-range contacts. The schematic diagram is designed to approximate the representation of the fold. The GNRA-like pentaloop structure is shown in yellow, A-form RNA helices are shown in blue and purple, the three-purine asymmetric bulge is in red, and the seven-nucleotide bubble is in green. Long-range tertiary contacts are indicated by thin red and yellow lines.

The virus that causes SARS, like other pathogenic coronaviruses and astroviruses, possesses a linear plus-sense strand RNA genome that has a 5′ methylated cap and 3′ poly-A tail. The viral replicase is translated directly from the genomic sense-strand RNA, and it then creates a full-length complementary (minus-sense strand) copy of the genomic RNA, as well as a nested set of shorter, subgenomic mRNAs having common 3′ UTRs. These 3′ UTRs all share with the genomic SARS RNA a 32-nucleotide element, immediately upstream of the 3′ poly-A tail (residues 29,590–29,621) [1], originally termed the stem-loop II motif (s2m) in human astroviruses [2]. The s2m element is the most highly conserved RNA element within the coronaviruses and astroviruses that contain it (Figure 1).

Standard structural genomics analyses focus upon obtaining the three-dimensional structures of proteins encoded within a genome, and on identifying unknown protein function based on three-dimensional homology to protein structures of known function [3]. However, it is also imperative to identify and to elucidate the three-dimensional structures of non-protein gene products, including the various RNAs required for mRNA processing, protein synthesis, and other cellular functions [4]. In the case of viruses that possess an RNA genome, including such pathogens as HIV and SARS, it becomes critical to expand the scope of structural genomics analyses even further to include biologically relevant RNA tertiary interactions that occur within the RNA genome itself. Those genomic RNA elements having the greatest degree of conservation are the most likely to be crucial to the evolution, growth, and replication of these viruses, and therefore demand the most attention from those seeking to understand RNA viral pathogenesis and to design appropriate anti-viral drugs.

Using X-ray crystallography, we have solved the three-dimensional structure of the SARS virus s2m RNA to 2.7-Å resolution. The structure reveals a dramatic 90° bend and several additional novel tertiary interactions. Although the sequence and three-dimensional structure of the s2m RNA are both unique, comparison of the global fold of the SARS s2m RNA to known RNA tertiary structures reveals that the backbone fold of the s2m RNA mimics that of the 530 loop of 16S rRNA, permitting us to hypothesize that the biological function of s2m in SARS and related viruses is based upon macromolecular mimicry of this region of ribosomal RNA. The ribosomal RNA 530 loop and the proteins that bind to it are involved in translational initiation, suggesting that the role of the s2m in SARS may also involve translation initiation. Specifically, we propose, based on structural homology arguments, that the SARS s2m RNA might bind to the host's eukaryotic translation initiation factor 1A (eIF-1A) to hijack the host's translational machinery for use by the virus, or to bind other translational regulation proteins having similar folds for similar purposes.

## Results

### Sequence Analysis of the Conserved s2m Element

We aligned the most recent available genomic sequences of coronaviruses and astroviruses and analyzed conservation patterns within the s2m element (Figure 1). Remarkably, about 75% of this sequence is absolutely invariant between viral species (nucleotides shown in boldface in Figure 1A) and much of the variation that does occur preserves secondary structural elements (nucleotides shown in italics in Figure 1A). In addition, we analyzed 38 sequenced SARS variants and found that the motif is absolutely conserved within all of them. No insertions or deletions appear to be tolerated, indicating that this region forms a highly conserved RNA tertiary structure that is universally required for viral function [1,2,5].

### The Crystal Structure of the s2m RNA Element of SARS

**Figure 2:**
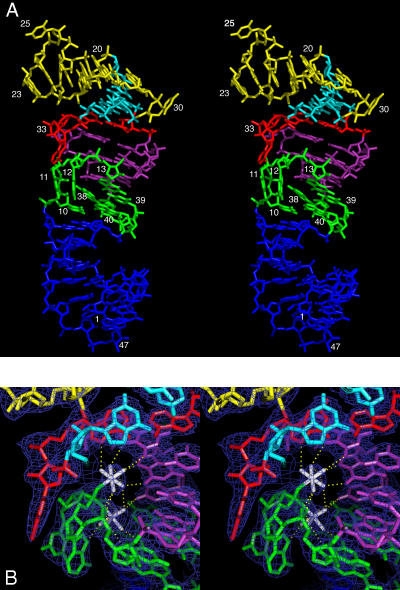
Stereo Representations of the SARS s2m RNA Structure (A) The overall SARS s2m RNA three-dimensional structure and (B) a detailed view of tertiary contacts the and [Mg(H_2_O)_5_]^2+^ binding sites in the context of the experimentally phased electron density map (dark blue). The [Mg(H_2_O)_5_]^2+^ complex ions, depicted as white octahedra, bind to the *pro*-R and *pro*-S phosphate oxygen atoms of A(12). An extensive network of potential hydrogen bonds between the metal-coordinated water molecules and the RNA is shown as yellow dotted lines.

Using in vitro transcription, we prepared and crystallized a 48-nucleotide construct containing the 45-nucleotide s2m element. We solved the crystal structure to 2.7-Å resolution using a single platinum isomorphous/anomalous derivative and obtained a readily interpretable solvent-flattened electron density map (Figures 1B–1D and 2A). The quality of the electron density enabled us to fit the s2m RNA sequence unambiguously to the map and to build a model of the unusual tertiary structure. The initial map was virtually indistinguishable from the final 3Fo–2Fc map calculated using phases from the refined RNA structure, indicating that the single isomorphous replacement with anomalous scattering (SIRAS) experimental phases initially obtained were quite accurate (Tables 1 and 2). Two well-ordered hydrated Mg^2+^ complexes bound to the phosphate backbone of the RNA are also readily observable in the initial electron density map (Figure 2B).

**Table 1:**
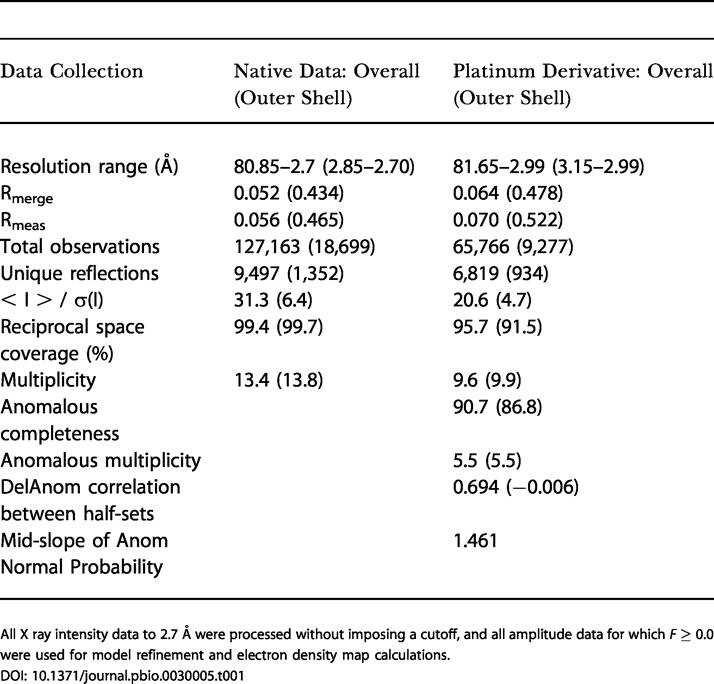
Crystallographic Data Collection

All X ray intensity data to 2.7 Å were processed without imposing a cutoff, and all amplitude data for which *F* ≥ 0.0 were used for model refinement and electron density map calculations

**Table 2:**
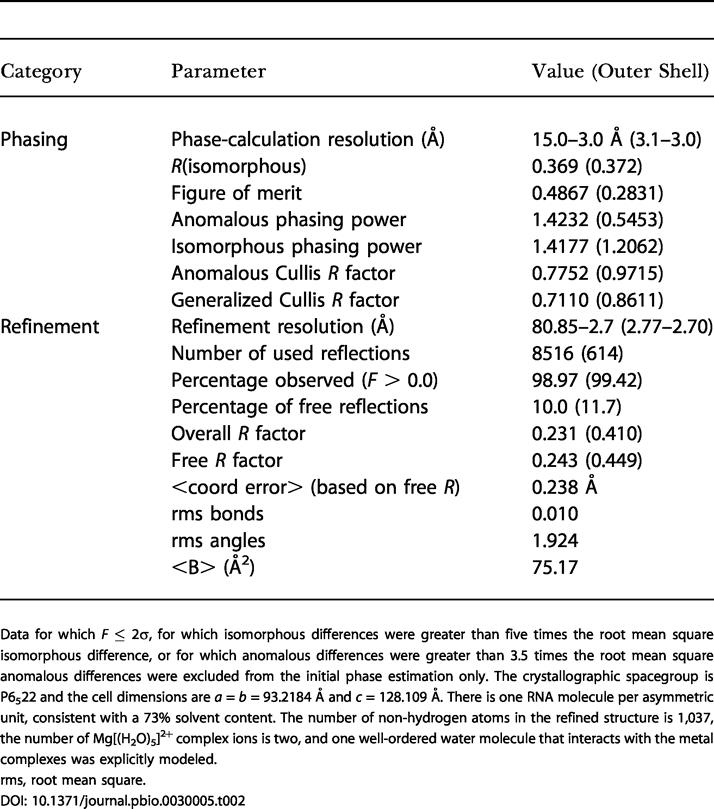
Phasing and Refinement

Data for which *F* ≤ 2σ, for which isomorphous differences were greater than five times the root mean square isomorphous difference, or for which anomalous differences were greater than 3.5 times the root mean square anomalous differences were excluded from the initial phase estimation only. The crystallographic spacegroup is P6_5_22 and the cell dimensions are *a* = *b* = 93.2184 Å and *c* = 128.109 Å. There is one RNA molecule per asymmetric unit, consistent with a 73% solvent content. The number of non-hydrogen atoms in the refined structure is 1,037, the number of Mg[(H_2_O)_5_]^2+^ complex ions is two, and one well-ordered water molecule that interacts with the metal complexes was explicitly modeled

rms, root mean square

**Figure 3:**
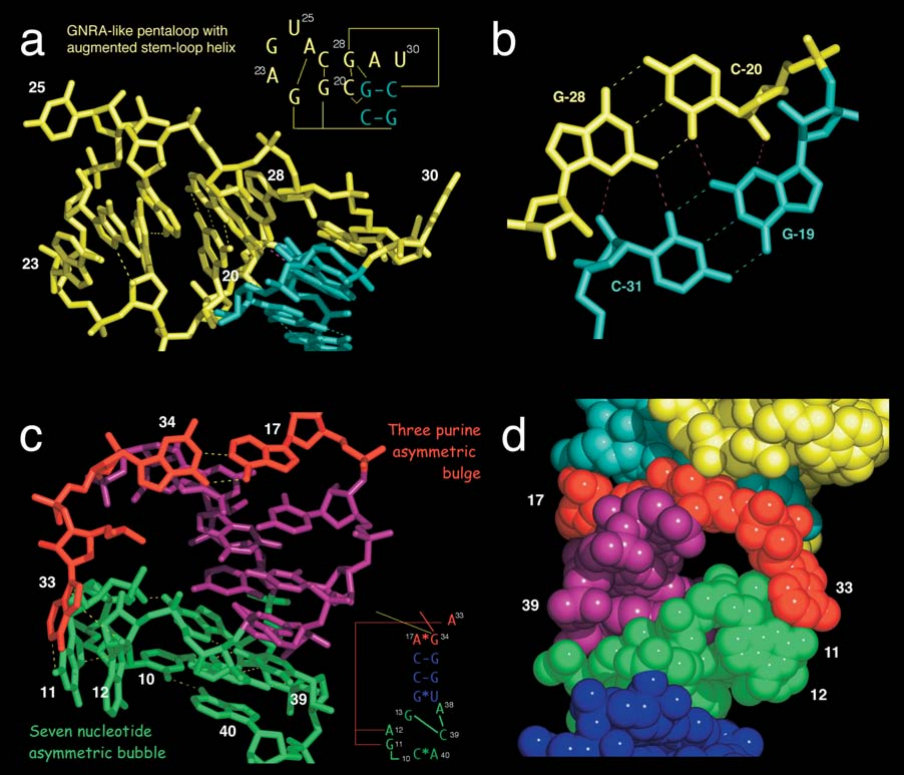
Tertiary Structural Interactions in the SARS s2m RNA (A) Close-up of the pentaloop structure together with the augmenting helix, shown in yellow, and the perpendicular junction formed with the A-form stem, shown in cyan. The pink hydrogen bonds indicate base-quartet hydrogen bonding, as shown in (B). The 90° kink thus formed is facilitated by a very sharp bend in the backbone involving unpaired residues 29 and 30. (B) Formation of the junction of two perpendicular helices is facilitated by a base quartet composed of two G–C pairs. (C) The unusual pairing between A(17) and G(34) facilitates formation of a long-range tertiary contact between A(33) of the three-purine asymmetric bulge and G(11) and A(12) of the seven-nucleotide asymmetric bubble. A(38) forms a base triple with C(39) and G(13), forcing G(11) and A(12) out of the main helix. (D) Space-filling representation of the region shown in (C), but rotated approximately 180°. A tunnel is created by the tertiary contacts between A(33) of the purine asymmetric bulge (red), G(11) and A(12) of the seven-nucleotide bubble (green), and the helical region between them (purple). The non-bridging phosphate oxygens of G(11) and A(12) line the surface of the cavity, creating a negatively charged region into which Mg^2+^ ions are observed to bind.

The crystal structure of the s2m domain of the SARS RNA reveals several novel tertiary structural elements (Figure 3). Three regions of canonical A-form RNA are indicated in various shades of blue, and three regions of unusual structure, including tertiary interactions, are represented in green, red, and yellow. The actual three-dimensional fold of the RNA is illustrated in Figure 1C, with Figure 1D designed to represent this fold schematically as well as the secondary and tertiary structural contacts that stabilize it. Figure 2A shows a corresponding stereo diagram in which all non-hydrogen atoms are present.

### The Fold of the s2m RNA, the Pentaloop, and a Nucleotide Quartet

The overall structure of the s2m SARS RNA consists of two regions that are defined by two perpendicular RNA helix axes (see Figures 1 and 2). The larger region contains several non-helical motifs involved in long-range tertiary contacts (see Figure 3). The smaller region (residues 20–30, shown in yellow in Figure 3) forms a stem-loop structure in which a pentaloop (residues 22–26) is structured similar to a conventional GNRA tetraloop motif but has an extra residue (U[25]) bulged out of the stack formed by A(23), G(24), A(26), and the augmenting helical stem (residues 20–21 and 27–28). This is similar to what is observed in a spliceosomal stem-loop structure [6]. The base of U(25) is disordered in the structure, and little side-chain density is apparent in an otherwise well-defined electron density map. Residues 29 and 30 are unpaired and are involved in forming a rather severe backbone reversal that accompanies the 90° kink in the helix axis. The phylogenetic comparisons shown in Figure 1A reveal that the pentaloop sequence is highly conserved. Although the structure of the pentaloop is very similar to the standard GNRA tetraloop structure [7,8], the “extra” U(25) insertion between R and A is always present. The unusual perpendicular helical junction is stabilized by the formation of an RNA base quartet involving two adjacent G–C pairs wherein the G(19)/C(31) pair shares four hydrogen bonds with the C(20)/G(28) pair (shown as pink dotted lines in Figure 3A and 3B). The RNA sequences required to preserve these G–C pair interactions are present in all but one of the viral sequences analyzed (avian nephritis virus), implying that the base quartet serves a significant structural role in SARS and most related viruses. All previously characterized RNA base quartets are purine tetrads [9,10,11,12,13,14,15] and do not occur within double-helical structures; the G–C quartet thus appears to be another novel structural feature present within the s2m element of SARS and related viruses.

### A Three-Purine Asymmetric Bulge

An asymmetric bulge in the s2m SARS RNA secondary structure containing A(17), A(33), and G(34) (highlighted in red in Figure 3C) is absolutely conserved in SARS and all other related viruses analyzed (as shown in Figure 1). A(17) pairs with G(34), involving the Watson–Crick base-pairing faces of both purines. This mode of interaction is rather distinct from the more usual “sheared” G–A pairings involving the Hoogsteen faces of these purines, and has the effect of significantly widening the RNA helix from the standard A-form geometry. As a consequence, A(33) is able to adopt a very unusual conformation in which it becomes completely excluded from the helical stack, and instead forms long-range tertiary interactions with G(11) and A(12). G(34), in addition to forming a Watson–Crick-like base pair with A(17), hydrogen bonds to C(18) as well as to G(21), thereby stabilizing the unusual pentaloop-stem conformation and 90° helical kink.

### A Seven-Nucleotide Asymmetric Bubble Interacts with the Purine Bulge

The remaining non-canonically base-paired region of secondary structure (residues 10–13 and 38–40), highlighted in green in Figure 3C, contains mostly conserved nucleotides including an absolutely conserved pair between C(10) and A(40), and a Watson–Crick pair within an otherwise highly distorted helical region between conserved residues G(13) and C(39). A base triple forms between A(38) and this G–C pair, a variant of the adenosine platform motif [16], and consequently G(11) and A(12) are rotated out of the helical structure completely. A(33) forms long-range tertiary interactions with G(11) and A(12) by hydrogen bonding to the N3 of G(11) and the ribose of A(12). Substitutions at position 12 are thus tolerated, as is a single instance of purine substitution at position 11 (which will preserve the N3 hydrogen-bonding interaction with A[12]). Together, these interactions superficially resemble those observed in domain IV of 4.5S RNA of the signal recognition particle [17,18], but the structural details are completely different.

G(11), A(12), and A(33), despite their extrusion from the helical base-pair stack, form a well-defined structure that is highly ordered, judging by electron density in the initial map as well as the comparatively low temperature factors these residues have in the refined structure. They conspire with the remaining residues in the asymmetric bubble and the helical region above it to form a rather wide tunnel whose channel runs approximately perpendicular to the main helical axis. The phosphates of G(11) and A(12) are turned inward, creating a negatively charged environment within the tunnel cavity. Consequently, the tunnel forms a binding site for two [Mg(H_2_O)_6_]^2+^ ions in the native structure (see Figure 2B), and the tunnel is also the binding site for *cis*-[(NH_3_)_2_Cl_2_Pt(IV)]^2+^ and [Ru(NH_3_)_6_]^3+^ metal complexes that were introduced for heavy atom isomorphous replacement phasing. These highly structured and rigorously conserved features allow us to suggest that SARS pathogenesis might be inhibited by a drug designed to bind to s2m and disrupt one of these structures.

### Chemical Probing of the Solution Structure

**Figure 4:**
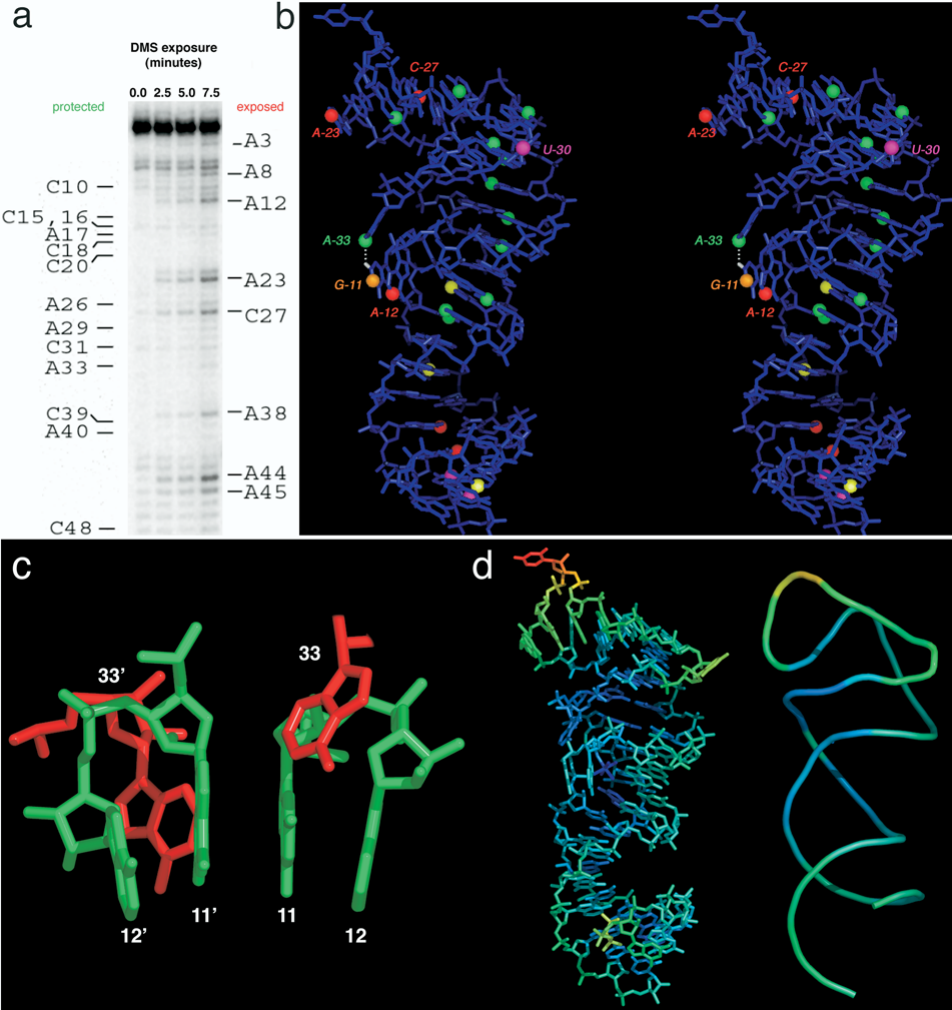
Chemical Probing of the SARS s2m RNA in Solution (A) An autoradiogram of DMS modification of the s2m RNA in solution. (B) Mapping the results of DMS, kethoxal, and CMCT modifications onto a stereo representation of the RNA structure. Red spheres represent strongly reactive N1 positions of adenosines and N3 positions of cytidine residues in the presence of DMS, and yellow spheres represent weaker reaction. Green spheres represent positions that appear to be protected from DMS. The orange sphere represents reaction with kethoxal at the N1 position of G(11), and magenta spheres represent CMCT reactions with uridines. (C) The most extensive crystal packing interaction involves stacking of G(11) upon its symmetry mate, G(11)′. (D) Temperature factors mapped onto all non-hydrogen atoms (left) and the phosphate backbone (right) of the s2m RNA crystal structure. U(25) is the most disordered residue in the structure and has the highest temperature factor. Density of the base of U(25) is not apparent even after refinement. Most of the rest of the structure is rather well ordered.

To compare the crystal structure with the solution structure of s2m, we performed chemical modification experiments. The results are consistent with the crystal structure, and in some cases enable us to verify that long-range tertiary interactions observed in the crystal structure also occur in solution. Dimethyl sulfate (DMS) modification patterns (Figure 4A) of the N1 atomic position of A and the N3 of C residues are consistent with the observed fold in the crystal structure (Figure 4B). A and C residues that are solvent-exposed in the tertiary structure, such as A(12), A(23), and C(27), are among the most heavily modified by DMS (along with A[44] and A[45] near the helical terminus). These modification sites are shown as red spheres in Figure 4B. Although A(33) is quite exposed in the tertiary structure, the N1 is protected from modification by DMS (shown as a green sphere in Figure 4B), consistent with the involvement of the N1 of A(33) in a 2.8-Å hydrogen bond with the exocyclic N2 of G(11) (white atom and dotted line in Figure 4B) in the crystal structure. We therefore conclude that this tertiary structural interaction observed in crystals of s2m RNA is likely to be quite similar to what occurs in solution. G(11) is the only G residue of the s2m RNA detectably modified by kethoxal (data not shown), which reacts with nitrogens at the N1 and N2 positions. The N1 modification site is highlighted as an orange sphere and is consistent with the observed tertiary structure formed by G(11), A(12), and A(33) that exposes G(11) to the solvent. U(30) is solvent-exposed in the crystal structure and is reactive to 1-cyclohexyl-3-(2-morpholinoethyl) carbodiimide metho-p-toluene sulfonate (CMCT; magenta spheres in Figure 4B; data not shown), as are the non-conserved 3′-terminal uridines (probably due to helix fraying in solution). U(25), which is not well ordered in the crystal but which we expect is also solvent-exposed, appears not to be reactive.

## Discussion

The intricate three-dimensional structure of the SARS s2m RNA, along with its rigorous sequence conservation, is compelling prima facie evidence for its biological importance in coronaviruses and astroviruses. The structure by itself, however, does not indicate what the function of this motif must be. Hence, comparison of this unique fold with those of known RNA structures is of particular value for formulating testable hypotheses regarding potential biological functions of the s2m RNA. In addition, identification of novel and rigorously conserved tertiary structures that are unique to the viral RNA is of critical importance for future rational design of anti-viral therapeutic agents that specifically target SARS and other coronaviruses and astroviruses.

### Biological Relevance of the s2m Sequence and Crystal Structure

The s2m RNA sequence we crystallized was originally identified from the genomic sense strand within a rigorously conserved region of the 3′ UTR of the RNA. However, because RNA replication and transcription take place via a full-length negative-strand RNA intermediate, it is formally possible that the conserved sequence instead corresponds to a conserved structure at the 5′ end of the anti-sense RNA. We believe this to be improbable because of the energetically unfavorable tertiary structures that would be required to form from the sequence complement. For example, the variant of the energetically stable and rather common GNRA loop structure (GAGUA) would have to be replaced with an energetically unstable and rare CUCAU loop. Similar arguments apply to the other non-Watson–Crick regions of the structure.

Crystal packing interactions may potentially distort RNA structures. This effect is sometimes observed for small stem-loop sequences, which often crystallize as duplex dimers rather than as monomeric hairpins. The s2m RNA structure is sufficiently large, and apparently contains enough stabilizing secondary and tertiary interactions, to offset any energetic advantage that might come from crystallizing as a duplex. In addition, the 73% solvent content of the s2m RNA crystals ensures that most of the crystallized RNA is solvent-exposed, rather than involved in extensive packing interactions. At least three inter-molecular contacts are required to form a crystal. The most extensive contact is the base of residue G(11); it stacks upon that of its 2-fold symmetry mate (Figure 4C). It is likely that these nucleotide bases become oriented in such a way as to optimize this stacking interaction. The nonessential nucleotide G(1) forms a weak (3.4-Å) hydrogen-bonding interaction with A(29) of an adjacent molecule, but most of this packing interaction appears to be due to shape complementarity and is thus expected to have little distorting effect. The remaining interaction is a nonspecific, presumably cation-mediated backbone parallel helical interaction, again unlikely to result in significant distortions.

Crystallographic temperature factors provide direct physical evidence for the relative flexibility or mobility of various regions of a macromolecule. Figure 4D shows relative temperature factors color-coded on all non-hydrogen atoms (left) and on the RNA phosphate backbone atoms (right). Blue atoms have the lowest relative temperature factors and red atoms have the highest. Consistent with the observed electron density map, by far the most flexible region of the RNA is U(25). U(30) and the 5′-terminal triphosphate are also moderately disordered. Much of the rest of the structure appears to be rather rigid and well defined, including the three-purine asymmetric bulge and the seven-nucleotide asymmetric bubble, along with the hydrated magnesium complex ions that bind to the non-bridging phosphate oxygens of A(12). The phosphate backbone atoms of these non-Watson–Crick regions are among the most ordered in the structure.

Therefore, based on our chemical probing data, analysis of crystal packing interactions, and consideration of the crystallographic temperature factors, along with the ability to rationalize the sequence conservation pattern and intolerance for nucleotide insertions or deletions based on the structure, we conclude that the crystal structure of s2m is likely to be a close representation of the structure that forms in solution and in the context of the SARS virus RNA genome.

### Functional Implications of the s2m Three-Dimensional Structure

**Figure 5:**
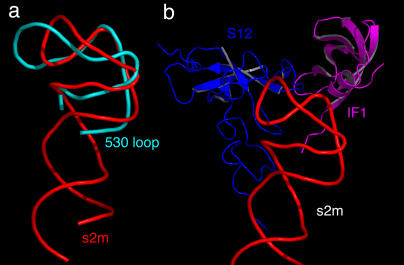
SARS Virus RNA Macromolecular Mimicry (A) The SARS s2m RNA structure (red) is superimposed upon the 530 loop of 16S rRNA (cyan), revealing the similar stem-loop folds. (B) The IF-1 (magenta) and S12 protein (blue) that bind to the 16S rRNA 530 loop (now hidden) are shown relative to the same s2m RNA superposition, suggesting that their eukaryotic homologs might plausibly bind to the s2m RNA.

The several unique features and unanticipated tertiary contacts we identified in the SARS s2m RNA crystal structure allowed us to reexamine genomic sequences and previously determined RNA tertiary structures for similar motifs with additional constraints imposed by knowledge of the tertiary structure. Our analysis of the human genome, other animal and viral genomes, and the currently available database of RNA three-dimensional structures revealed that the s2m element is found only in astroviruses and coronaviruses; no cellular homologs are immediately apparent. The G(11) to A(33) tertiary contact in the s2m RNA is homologous to the G(1,452) to A(1,486) contact in Domain III of the 23S ribosomal RNA, but the context of the interaction in the ribosome is completely different, and the sequence is not conserved between Escherichia coli and Thermus thermophilus. However, if we relax the sequence constraints and focus attention upon the conformation of the RNA backbone, we find that the phosphodiester backbone fold accompanying the 90° kink in s2m RNA mimics that found in the 530 stem-loop of 16S ribosomal RNA [19] (Figure 5A). The latter binds to the S12 protein found at the interface between the small and large ribosomal subunits. The 530 stem-loop, and the S12 protein that binds to it, have been implicated in EF–G-independent ribosomal translocation [20]. Remarkably, superposition of the s2m RNA upon the 530 stem-loop within the 30S ribosome in which prokaryotic initiation factor 1 (IF-1) has been added [21] reveals plausible modes of s2m RNA binding to both the S12 protein and to IF-1 (Figure 5B). Both S12 and IF-1 have eukaryotic homologs; the structure of IF-1 and its eukaryotic analog, eIF-1A, possess almost identical RNA oligomer binding (OB) folds [22,23]. Based upon these structural homology arguments, we propose that the SARS s2m RNA is a functional macromolecular mimic of the 530 loop of the small subunit ribosomal RNA (which is conserved in eukaryotes). Mechanisms of translation and protein synthesis regulation via macromolecular mimicry are in fact well established [24,25]. We propose, on the basis of the similarity between the 530-loop fold and the s2m fold, that the s2m RNA of SARS may be capable of binding one or more eukaryotic proteins whose structures resemble S12 or the OB folds typical of these ribosomal proteins, and that each would do so in a manner similar to that shown in Figure 5B. This proposal leads us to formulate two separate, testable hypotheses regarding the function of the s2m RNA in SARS.

### Does s2m Macromolecular Mimicry Facilitate Viral Hijacking of Protein Synthesis?

eIF-1A, like IF-1, possesses an OB fold. Our first hypothesis is that eIF-1A may bind to the 90° bend of the SARS s2m RNA. In addition, we suggest that the function of the s2m RNA of SARS and related viruses might involve viral hijacking [26] of the cell's protein synthesis machinery, either facilitating mRNA circularization and ribosome re-initiation, in gross analogy to viral internal ribosomal entry site–mediated mechanisms [27,28], or perhaps even more simply by titrating eIF-1A away from the host initiation complexes and thus inhibiting host cell protein synthesis in favor of viral protein synthesis by sequestering a factor required by the host.

### Does s2m Bind to the nsp9 SARS Protein to Facilitate Virus Transcription?

Recently, two protein structural genomics investigations of SARS revealed the structure of a so-called nonstructural protein, nsp9, that is believed to be involved in viral RNA synthesis and to interact with the viral polymerase in an unspecified manner [29,30,31]. The crystal structure of nsp9 reveals it to be a variant of the OB fold, a protein structural motif not previously recognized to be involved in viral replication. The authors demonstrate nonspecific single-strand RNA binding affinity for nsp9. We propose that nsp9, by virtue of its OB fold, may bind specifically to s2m in a manner similar to that illustrated in Figure 5B, and may thus facilitate viral polymerase RNA transcription, translation, or replication.

### From Structure to Functional Predictions

Our structural genomics analysis of the SARS RNA has thus enabled us to formulate specific, experimentally testable hypotheses regarding the function of a highly conserved RNA motif whose importance has been evident [2] but whose biological activity hitherto was completely unknown. The possibility that the 90° bend of the s2m RNA binds to an OB-like protein permits us to propose two potential mechanisms of interaction relevant to the two main functions of the SARS virus (protein synthesis and viral replication). The possibility of additional interactions with proteins at the S12-like site and in the highly structured and rigorously conserved tunnel region formed by the three-purine bulge and the seven-nucleotide bubble should also not be overlooked, as these both are likely sites for RNA–protein or RNA–RNA interactions that are crucial to the function of the SARS virus, and therefore also merit further attention.

### The s2m RNA Tunnel Is an Attractive Target for the Design of Anti-SARS Drugs

Figure 3C and 3D dramatically illustrates the most striking and unique structural feature within the SARS s2m RNA. A tunnel is created by the tertiary contacts between A(33) of the purine asymmetric bulge (red), G(11) and A(12) of the seven-nucleotide bubble (green), and the helical region between them (purple). The non-bridging phosphate oxygens of G(11) and A(12) line the surface of the cavity, creating a negatively charged region into which Mg^2+^ ions are observed to bind. It is likely that in the context of the virus, this invariant feature of the s2m structure is involved in binding interactions with highly conserved proteins or other components of the host cell that interact specifically with the negatively charged cavity. Because this tunnel structure is unique to coronaviruses and astroviruses and because the sequence comprising this structure is invariant, it is reasonable to propose that by designing a drug that specifically targets this structural feature and binds tightly to it, an anti-SARS therapeutic might be obtained that avoids the pitfall of being toxic to uninfected host cells while escaping the usual problem of drug resistance that develops in rapidly mutating RNA viruses.

## Materials and Methods

### 

#### 

Crystals of a 48-nucleotide T7 RNA transcript containing the conserved s2m RNA element were obtained via hanging-drop vapor diffusion by equilibrating a solution containing equal volumes of the RNA sample and the reservoir solution against 1-ml of the reservoir solution. The RNA sample solution contained 4.5 mg/ml s2m RNA dissolved in 30 mM Tris (pH 7.6), 100 mM NaCl, and 60 mM MgCl_2_. The reservoir solution contained 50 mM MES (pH 5.6), 100 mM Mg(OAc)_2_, and 20% MPD. Data from a native crystal diffracting to 2.7-Å resolution, and 3.0-Å *cis*-(NH_3_)_2_(Cl)_2_Pt(IV)–derivative single-wavelength anomalous dispersion data, were collected at Beamline 9.1 at Stanford Synchrotron Radiation Laboratory on a 3 × 3 CCD detector using 0.98-Å wavelength X rays and crystals that were cryoprotected in the reservoir solution spiked with 12% glycerol and maintained at 100 K. The native and platinum derivative data were processed using CCP4's MOSFLM and reduced and scaled within CCP4 version 5.0 [32,33]. A single platinum heavy atom site was found in both isomorphous- and anomalous-differences Patterson-map Harker sections calculated using data from 10- to 5-Å resolution. Phase calculation, solvent flattening, phase extension, and simulated annealing refinement were carried out within CNS version 1.1 [34]. The initial SIRAS map was uninterruptible in spacegroup P6_1_22 but was unambiguous in P6_5_22, permitting the hand of the space group to be determined. A 47-nucleotide poly-C model was built into the SIRAS map using O, the actual nucleotide-sequence register was then confirmed by inspecting the electron density, and residues 1–47 were built in using O [35]. The phosphate for residue 48 is clearly present in the electron density map, but the density for the remainder of U(48), as well as that for the bases of U(25) and U(30), was rather disordered. The final refinement was performed using CCP4's refmac [36], and the figures were produced using MacPymol [37]. All crystallographic computations were performed on the Mac OS X platform. Details of data processing, phasing, and refinement are provided in Tables 1 and 2. The crystal structure of the SARS s2m RNA was compared to others in the RCSB Protein Data Bank using the program MC-Annotate [38,39] and by visual inspection. Sequence comparisons prior to obtaining the s2m tertiary structure were performed using the UCSC Genome Browser [40], and were subsequently supplemented with tertiary constraints imposed by the crystal structure using the programs PatScan [41] and RNABOB [42,43]. Transcripts containing s2m for solution structure analysis were prepared using plasmid templates cleaved downstream, so that the s2m element was present at the 5′ end of the transcript and contained an RNA tail consisting of plasmid sequences. Chemical probing experiments were carried out according to established methods [44]. Primer extension was performed as described previously [45] using a primer complementary to sequences 3′ of the s2m element.

## Supporting Information

Coordinates, native and derivative amplitudes, and experimental phases have been deposited in the RCSB Protein Data Bank (http://www.rcsb.org/pdb/) under accession number 1XJR and are also available with other supplementary materials at http://www.chemistry.ucsc.edu/%7Ewgscott/sars.

### Accession Numbers

The RCSB Protein Data Bank accession number for the SARS s2m RNA structure reported here is 1XJR. The RCSB Protein Data Bank accession numbers for the other protein and RNA structures discussed in this paper are as follows: the 30S ribosome (1J5E), the 30S ribosome in which prokaryotic IF-1 has been added (1HR0), the eukaryotic analog of prokaryotic IF-1 (1D7Q), and the crystal structure of nsp9 (1QZ8 and 1UW7).

## Abbreviations

CMCT: 1-cyclohexyl-3-(2-morpholinoethyl) carbodiimide metho-p-toluene sulfonate
DMS: dimethyl sulfate
eIF-1A: eukaryotic initiation factor 1A
IF-1: initiation factor 1
OB: oligomer binding
s2m: stem-loop II motif
SIRAS: single isomorphous replacement with anomalous scattering
